# Psychotropic Medication Informed Consent: A Cross-Specialty Role-Playing Skill Builder

**DOI:** 10.15766/mep_2374-8265.11152

**Published:** 2021-05-05

**Authors:** Emily Diana, Derrick Hamaoka, Matthew Goldenberg, Kelly L. Cozza

**Affiliations:** 1 Fourth-Year Medical Student, Uniformed Services University of the Health Sciences F. Edward Hébert School of Medicine; 2 Associate Professor, Department of Psychiatry, Uniformed Services University of the Health Sciences; 3 Associate Professor, Department of Psychiatry, Yale School of Medicine; 4 Professor, Department of Psychiatry, Uniformed Services University of the Health Sciences

**Keywords:** Informed Consent, Entrustable Professional Activity 11, Role Play/Dramatization, Role-Playing, Shared Decision-Making, Communication Skills, Competency-Based Medical Education (Competencies, Milestones, EPAs)

## Abstract

**Introduction:**

Obtaining informed consent (IC) is an essential medical practice. Utilization of IC role-playing training with medication study cards and self-peer-supervisor review should improve student fund of knowledge and strengthen IC skills for clerkship-level medical students.

**Methods:**

Between 2017 and 2020, approximately 555 clerkship medical students used our formative role-playing exercise tools. Students independently prepared psychotropic medication study cards and role-played IC during group didactics. Peer and supervisor reviews were not recorded but were discussed as a group. Students completed routine anonymous postclerkship surveys regarding the IC exercise. An enhanced IC curriculum was deployed in 2020, adding a training video and peer/supervisor feedback form. Student feedback and specialty shelf exam scores were reviewed to assess the exercise's effectiveness.

**Results:**

Surveys indicated satisfaction with the exercise and increased confidence in obtaining IC. Interestingly, the student group that received enhanced IC training had fewer shelf exam failures than those without, perhaps indicating improved fund of psychotropic medication knowledge.

**Discussion:**

Peer role-playing IC training is well accepted by students, allows practice of essential elements of IC and shared decision-making, and provides an engaging way to improve medication fund of knowledge. Our clerkship has initiated development of an IC objective structured clinical examination station and is adapting the exercise across specialties for longitudinal learning in response to the positive feedback and ease of use. Structured review of psychotropics and peer IC role-playing can be tailored for other specialties, medications, and procedures and further developed for use in pre- and postclerkship education.

## Educational Objectives

By the end of this activity, learners will be able to:

1. Memorize and utilize an essential steps rubric for obtaining informed consent.
2. Develop and demonstrate psychotropic medication fund of knowledge.
3. Propose and discuss a medication plan in patient-friendly terms.
4. Practice shared decision-making techniques.
5. Assess and discuss self- and peer performance in obtaining informed consent.

## Introduction

Discussing treatment options and obtaining informed consent (IC) are cornerstones of medical practice. Physicians have an ethical and legal duty to participate in IC-covered, shared decision-making conversations with patients to allow them to make knowledgeable and more autonomous medical decisions.^1,2^ According to the Association of American Medical Colleges, obtaining IC for tests and/or procedures is regarded as one of the core Entrustable Professional Activities (EPAs), EPA 11, meaning that medical students should be proficient at this activity before entering residency.^3^ The incorporation of IC practice into medical student learning has been found to be effective by both medical students and patients.^4,5^ To address EPA 11, the Uniformed Services University (USU) School of Medicine developed an IC curriculum for clerkship students using rubric-driven medication study cards with peer- and faculty-reviewed patient vignette role-playing to practice this skill. This exercise was developed to prepare students for clinical practice and written and oral examinations.

A lack of uniformity among IC curricula for both students and residents may lead to varying degrees of comfort in obtaining IC, with the majority of residents reporting learning by observation in residency.^6–8^ A survey of medical providers including third- and fourth-year medical students found that 60% of participants felt their IC training was inadequate, with 35% experiencing difficulty obtaining IC.^9^ Additionally, the quality of IC practice impacts patients’ abilities to make informed decisions about their health. A study found that up to half of patients after a procedure did not recall the explanation of risks and up to 90%, depending on specialty, did not recall a discussion of alternatives.^10^ Though the skill of obtaining IC is identified as a core EPA for physicians, there is currently no standardized method to teach, assess, or track competency for students preparing to enter residency.^11^ This gap in education is not lost on medical students, who perceive the importance of IC and, overall, want more IC training.^12,13^ By targeting medical students during their clerkship rotations, a standardized IC curriculum gives providers a baseline on which to cultivate their IC practices.

Though there are no uniform teaching or assessment methods across medical schools, techniques such as standardized patient encounters and objective structured clinical examinations (OSCEs) have been used selectively to teach or assess students in the process of IC.^14–16^ Some educational models focus on IC in the context of health literacy or bioethical perspectives, using case examples and review questions.^17,18^ Other models use standardized patients with feedback to specifically target senior medical students or early residents and assess IC abilities for procedural cases.^17,19^ Residency-level IC training is available for standardized procedures such as blood transfusion or CT guided lung biopsy.^20,21^

Our USU IC exercise was designed to offer students a low-stakes environment in which to practice the fundamentals of obtaining IC, as well as provide them with peer and supervisor feedback. This exercise is unique given the ease and accessibility of role-playing, which can be performed anytime or anywhere among students in person or via distance learning. Additionally, preparing rubric-driven IC and medication study cards provides a structured and engaging approach to improve students’ clinical fund of knowledge and doubles as a preparation method for written and clinical examinations. The technique of teaching IC by vignette role-playing can easily be extrapolated for use across other specialties and for procedures and can be adapted to assess competency over time.

## Methods

USU psychiatry clerkship students in their 18th to 30th months of undergraduate medical education were the learners for the IC role-playing exercise. An approximate total of 555 students participated in the exercises between January 2017 and April 2020. During the 5-week psychiatry clerkship rotation, we provided student instructions for the exercise (Appendix A) and 25 brief vignettes of patients in need of psychotropic medication (Appendix B). We tasked students with completing five IC/medication study cards per week in preparation for didactics, which took place either in person or remotely via a videoconferencing platform based on student clerkship location (Appendix C).

Preceptors, ward attendings, residents, and weekly didactic faculty received these vignettes and additional faculty instructions (Appendix D). Students observed a faculty member modeling an IC role-play vignette during the first week of clerkship training. In subsequent weeks, students volunteered or were randomly selected to role-play in front of their peers and supervisors, with one student in the role of patient while another played the provider obtaining IC in order to practice shared decision-making. Preceptors timed the role-play from start to finish, with a goal of less than 15 minutes for completion of the exercise by the end of the rotation.

We introduced an enhanced curriculum in January 2020 based upon anonymous student feedback, adding a faculty-developed peer/supervisor feedback form (Appendix E) that followed the IC/medication study card format and providing a faculty and role-playing actor video example of IC shared decision-making (Appendix F).

The rubric-style feedback form included medication indication, mechanism of action, duration of use, dosing schedule, common side effects, black box warnings, possible toxicities, contraindications, alternative treatments, course of illness without medication, and emergency contact instructions. Since this exercise was designed to be formative learning, feedback forms were shared with the students but not collected or recorded.

Observing students and faculty used the peer/supervisor feedback form to provide immediate formative feedback regarding required elements of IC and also discussed fluency by focusing on sections 4–6 of the Essential Elements of Communication (EEC) checklist (Appendix G),^22^ a learning tool used throughout clerkship training. Though a crucial part of IC, assessing decisional capacity and documentation of IC was not conducive to this brief role-playing exercise, and since these elements were taught elsewhere in the curriculum, they were not explicitly taught or included during this exercise.

Throughout 2019–2020, we evaluated the utility of the IC exercise with student feedback using a routine anonymous survey with 5-point Likert scales and open-ended feedback (Appendix H). Bolus noncontinuous survey feedback was obtained crossing academic years (AYs) 2018–2020. Institutional review board exemption was granted to explore student survey and shelf exam data retrospectively in order to understand the impact of the exercise on student satisfaction and medication fund of knowledge.

USU psychiatry clerkship student USMLE NBME Psychiatry Specialty Exam results were reviewed for classes without IC role-playing experience (AY 2016, *N* = 170), classes with students who had completed first-edition IC role-playing (AYs 2017–2019, *N* = 515), and classes with students completing the enhanced IC training in the first two 5-week rotations of AY 2020 (*N* = 40).

## Results

### Student Surveys

IC-specific anonymous student feedback surveys were sent in boluses in 2019 and again in 2020. We received 63 survey responses in total (see Table 1 for details). Fifty-nine percent of all student respondents indicated that no other rotation explicitly taught IC. When asked about the practicality of the exercise, 83% of learner respondents found that the selection of medications and procedures for the role-playing exercise was appropriate. Overall, students found the exercise beneficial, with 60% reporting increased comfort in obtaining IC while on the psychiatry service as well as 59% while on other services. Students reported that performing this skill in clinical practice would have boosted the exercise, as only 54% were able to practice IC with clinical patients during the clerkship. Seventy percent of students indicated that they prepared for the exercise in advance, with 44% of students finding the exercise helpful in preparation for standardized NBME examinations.

**Table 1.**
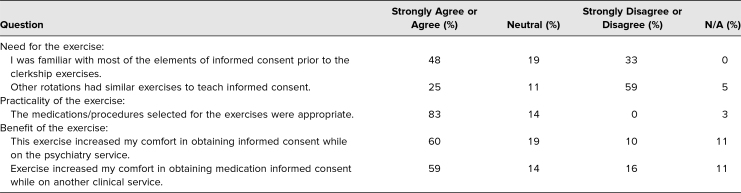
Anonymous Informed Consent Exercise Student Survey Results (*N* = 63)

Students noted the strengths of the IC role-playing exercise in the open-comment section of the survey, including comments about the uniqueness of the exercise among all clerkship curricula. Common themes also included the exercise's impact upon improving psychotropic medication familiarity and comfort with the IC process.

Learner feedback concerning exercise strengths included the following examples:

• “Practical education of an extremely important topic that is not often explicitly taught.”
• “Only time I got to practice informed consent on Psych, and one of very few times during Clerkship year.”
• “Role playing enhanced my ability to remember facts about the meds.”
• “It introduces us to the concept of informed consent and prepares us for clinical responsibilities that increase over time. I thought more about the side effects of medications as a result of the exercise and I had to learn the tools and resources to find this information.”
• “Perfect practice makes perfect. Having real time feedback on how we performed, and then repeating this process weekly helped to solidify the basic principles of medication consent.”
• “It wasn't too formal, which takes pressure off of those who may be nervous speaking in front of a group. Feedback was always positive and then we contributed as a group to fill in gaps a classmate may have missed. The collective nature was great.”

Overall themes for needed improvement included shortening the exercise and providing more standardization between preceptors. Additionally, several learners with in-person didactics suggested pairing up or assigning medications for role-playing to allow each student more opportunity to practice. Generally, there was the same number of learners who approved of the exercise as there was of those who thought the exercise was too long.

Learner feedback about the need for exercise improvements included the following examples:

• “I felt the informed consent during the exercise was more in depth than what actually occurs in real time. I would suggest tapering the exercise to highlight the most relevant points of informed consent.”
• “A completed example would have been helpful. Not having done it before, it was confusing to try to figure out what information (side effects) was actually important to include. It wasn't until I watched an informed consent that I actually understood what information was important.”
• “Not sure it needs to be a part of the OSCE. I feel as though I got good feedback from my sessions and it made me aware of some of the challenges associated with IC. I asked in my own clerkship to do an informed consent when a patient was started on a new med and I found that incredibly useful. I am not sure how much I would get out of doing it in front of a camera with a sim patient who has memorized a standardized script.”
• “The list of drugs was extensive each week in addition to all the other material we had to study for. I would cut the list down in order to make it more manageable. Additionally, we were not held accountable for the information in our lectures or small groups. This limited preparation and full buy in to the assignment.”

### Assessment

Overall NBME results for students without IC role-playing experience (AYs prior to 2017) and those with first-edition IC role-playing (AYs 2017–2019) revealed no differences in performance (Table 2). For AY 2020, the exam results of the student group that received enhanced IC training trended toward fewer NBME failures (5% for AY 2020, *N* = 40) in the first two rotations of the academic year than students who completed the first-edition role-playing IC exercise in the first two rotations of previous years (14% for AY 2017, *N* = 59; 15% for AY 2018, *N* = 59; and 16% for AY 2019, *N* = 55).

**Table 2.**
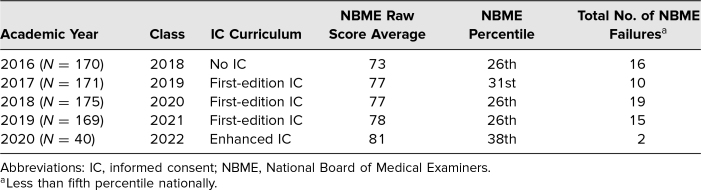
Scored Clerkship Assessment Data

## Discussion

IC role-playing exercises provide an easy-to-use, low-stakes, formative learning opportunity for the essentials of obtaining IC, while offering a structured approach to learning specifics about psychotropic medications. Perhaps the most crucial benefit of the IC role-playing exercise is demonstrated by our students’ subjective increase in confidence in discussing medication use with patients on both psychiatry and other clerkships. Additionally, while preparing for the exercise, students are able to learn testable facts and concepts to use while obtaining IC, as evidenced by improved Psychiatry Subject Examination results in this pilot sample. The IC training exercise provides a simple and innovative way for students to learn about and practice obtaining IC, which supports the objective for EPA 11. Implementation is fast (<30 minutes for each student), easy to include in existing didactics, and flexible for in-person or remote/virtual learning. Furthermore, the exercise can be expanded to faculty-resident and resident-student applications in a longitudinal approach and is adjustable for inclusion in other specialties for practice with medications and procedures. The ease of the exercise and its potential for expansion to other specialties or trainee levels need further experience but are easily translatable.

Our IC exercise has evolved since its launch in 2017 by incorporating student feedback, which influenced the addition of the IC video example, peer/supervisor feedback form, and modified EEC checklist (Appendices E–G). Importantly, student use of the peer/supervisor feedback form as a guiding checklist ensures students incorporate all components to include the essential element of shared decision-making and provides meaningful, formative comments from peers and faculty concerning strengths and weaknesses. The EEC checklist aids in the refinement of communication skills needed when obtaining IC in a shared decision-making manner.

Our survey highlighted the need for an IC exercise in the psychiatry clerkship curriculum since less than half of our students were familiar with the elements of IC prior to this training (Table 1). Overall, students found that the IC role-playing added to their learning and filled a gap in the curriculum, as no other specialty explicitly taught this important element of clinical medicine. Open-ended survey comments suggested that students found the exercise helpful and appreciated receiving prompt feedback on their performance. The increased comfort in obtaining IC for psychotropic medication likely indicates that students improved in skills such as speed, fluency, and confidence. We anticipate that students, armed with the tools of improved communication abilities and a better understanding of the components of IC, will advance even further in IC skills as they continue their undergraduate and residency education.

The NBME scores were not significantly different between the without-IC curriculum group and the first-edition IC curriculum groups, which may be possibly due to the lack of uniform guidelines for students. Variability among facilitators may have resulted in students receiving emphasis on learning some IC elements over others or having a focus on communication fluency rather than the required elements themselves. We attempted to mitigate this challenge in the enhanced curriculum through development of the peer/supervisor feedback form to streamline the elements of the IC for students to learn. In addition, a completed video example of obtaining IC, showing all the required elements, was distributed across all learners regardless of facilitator to improve uniformity of the instruction. Though we have a low suspicion for the alternative possibility, it is conceivable that the NBME scores may not have improved regardless of type of IC curriculum and that the score improvement with the enhanced IC group may have resulted from necessary alterations to curriculum and testing as a result of a global pandemic during the same time frame.

We feel it is likely the improved NBME scores for the students in the enhanced IC training group derived from the improved fund of knowledge as a result of emphasis on the preparation of medication study cards. Based on the NBME breakdown^23^ of the psychiatry shelf exam, approximately 30%-35% of the exam content concerns pharmacotherapy, intervention, and management, with approximately 60%-65% of questions reflecting an ambulatory setting, similar to the IC exercise setting. While this finding may also be attributed to other factors of the course, it is reasonable to conclude that the IC exercise impacted positively upon students’ fund of knowledge and their testing performance.

There are limitations to the generalizability of our results. Firstly, though instruction was provided to all facilitators prior to starting the exercise, feedback showed high variability among facilitators based on site location and week. Secondly, there was a low response rate for survey feedback, which was only collected for a portion of our students exposed to the role-playing exercise. Finally, the enhanced IC curriculum was deployed for only a small cohort of students in their first months of clerkship during a pandemic, which added several confounding variables, including the need to pivot all student learning to the virtual environment. Additionally, due to the success of the preenhanced curriculum, the clerkship deployed an IC OSCE station that had been set for March 2020 but was delayed and impacted by the COVID pandemic. The pending IC OSCE station assessment may have added an element of pressure and incentive for these students to participate and focus on the exercise to a greater extent than their predecessors. We continued to deploy the IC OSCE station virtually, and we will be reporting upon that assessment model at a later time after the delayed AY is completed. Further study is needed to assess whether the exercise translates to students successfully obtaining IC in a simulated environment.

Our USU clerkship will continue to utilize this IC role-playing exercise as part of our clerkship curriculum. We hope to begin introducing IC preparation in the preclerkship curriculum, with exposure to the medication study card prep and watching the IC video. During the clerkship time, expansion to multispecialty and resident-student dyads, as well as supervised real-patient IC experiences, is in progress. This interactive role-playing exercise can also be expanded with higher-level goals and objectives to include assessment of decisional capacity and documentation of the interaction, skills that may be well suited to a full simulated patient interview instead of a short vignette role-play. We have found this simple IC/medication preparation and role-playing exercise to be an easily deployable and enjoyable formative learning experience that assists in preparing our students to meet the expectations of EPA 11, demonstrating the ability to obtain IC.

## Appendices

Student Instructions.docxVignettes.docxIC & Medication Study Card Instructions.docxFaculty Instructions.docxPeer & Supervisor Feedback Form.docxExample.mp4Essential Elements of Communication.pdfStudent Survey.docx
All appendices are peer reviewed as integral parts of the Original Publication.
