# Supplementary material for: Psychotropic Medication Informed Consent: A Cross-Specialty Role-Playing Skill Builder
Source: MedEdPORTAL. 2021 May 5;17:11152. doi: 10.15766/mep_2374-8265.11152 (PMC8096884; doi:10.15766/mep_2374-8265.11152)
Supplement: Supplementary file 1 — Student Instructions.docxVignettes.docxIC & Medication Study Card Instructions.docxFaculty Instructions.docxPeer & Supervisor Feedback Form.docxExample.mp4Essential Elements of Communication.pdfStudent Survey.docx [file mep_2374-8265.11152-s001.zip › A. Student Instructions.docx]

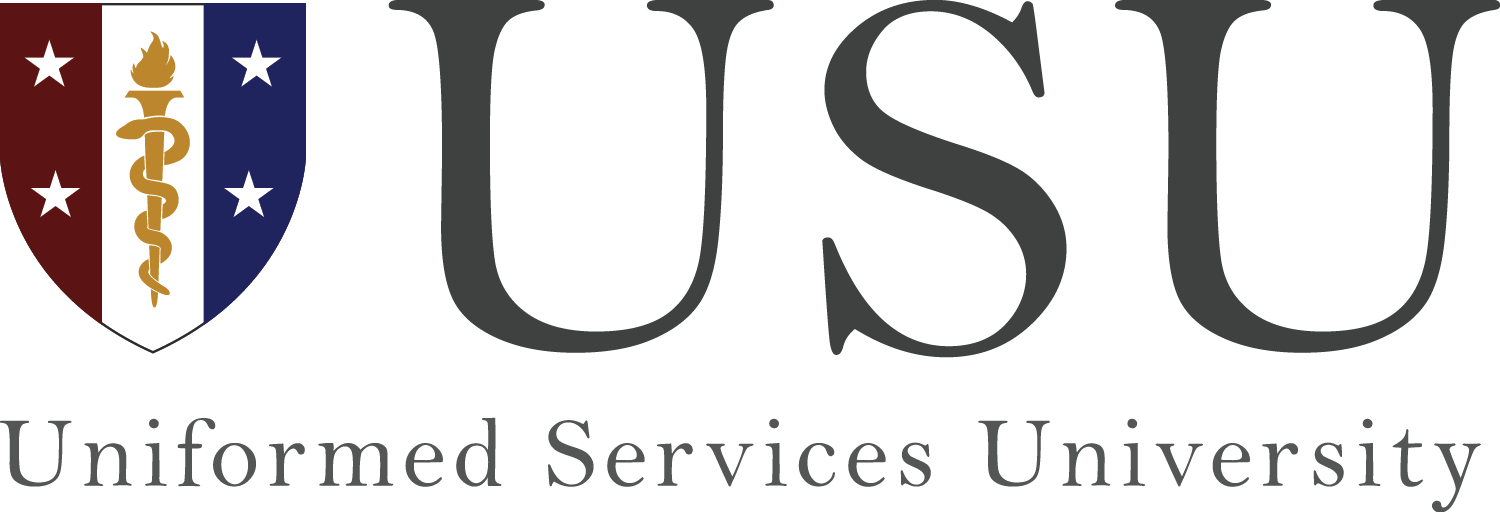


**Psychiatry Clerkship**

**Weekly Didactics**

**INFORMED CONSENT EXERCISES**

Last update: Feb 2021

Disclaimer: The opinions and assertions contained herein are those of the authors and do not reflect those of the Uniformed Services University or the Department of Defense.

**Informed Consent Exercise**

**Student Role-play Instructions**

Objectives:

1. Memorize and utilize an essential steps rubric for obtaining Informed Consent

2. Develop and demonstrate psychotropic medication fund of knowledge

3. Propose and discuss medication plan in patient-friendly terms

4. Practice shared decision-making techniques

5. Assess and discuss self and peer performance in obtaining informed consent

This activity is designed to help you develop and to improve your ability to obtain informed consent (IC) for medications with patients as well as help prepare you for SHELF/OSCE exams. Being able to explain complex medications and side effects to patients is paramount. Preparing for these cases yourself has the “side effect” of making great study sheets.

This exercise focuses on specific aspects of obtaining informed consent: presenting information about the recommended treatments and engaging in shared decision making. Please remember that the entire informed consent process involves assessing patient understanding, sharing information about the recommended treatment(s), engaging in shared decision making, and documenting the discussion. This exercise assumes the full assessment has already occurred, and that documentation will happen later.

Prior to class each week, prepare a Medication Study Card (see Medication Study Card Instructions) for each medication listed in the weekly IC vignettes. During each exercise, two students will be selected to role-play obtaining informed consent from a faculty member or another student who will play the role of the patient described in the vignette. Students not selected to role-play should fill out the peer/supervisor feedback form and essential elements of communication (EEC) form for the observed encounter.

As you get better at learning the CONTENT is needed in obtaining informed consent (the stuff on your study card), we will start to focus on HOW to obtain informed consent in a collaborative and easy style. Things to consider:

- What words should you use? Taper versus decrease? How to alter for different patients.
- What should the patient do for the side effects that you describe? Call you? Call 911? Watch and wait?
- When will the patient see you again?
- What side effects / labs / etc. will YOU, the doctor, be watching out for, and how do you include them in the watching?
- Practice shared decision-making…Asking patient what they think/know and engage them in the process
- Practice “teach back” by asking the patient to repeat or teach you what you have described to them
- Develop a clear side-effect/emergency/follow up plan that is understood by the patient
