# Supplementary material for: Psychotropic Medication Informed Consent: A Cross-Specialty Role-Playing Skill Builder
Source: MedEdPORTAL. 2021 May 5;17:11152. doi: 10.15766/mep_2374-8265.11152 (PMC8096884; doi:10.15766/mep_2374-8265.11152)
Supplement: Supplementary file 1 — Student Instructions.docxVignettes.docxIC & Medication Study Card Instructions.docxFaculty Instructions.docxPeer & Supervisor Feedback Form.docxExample.mp4Essential Elements of Communication.pdfStudent Survey.docx [file mep_2374-8265.11152-s001.zip › B. Vignettes.docx]

**Informed Consent Exercise Week 1**

**Fluoxetine (Prozac)**

Patient is a 22-year-old man with a recent onset of severe anxiety including increasing worries about contamination. He was recently put onto probation at work because of frequent tardiness, which he attributes to not being able to get “clean enough” in the mornings. He has never taken psychiatric medication previously.

**Nortriptyline (Pamelor)**

Patient is a 40-year-old woman with a history of chronic migraine headaches, back and genitourinary pain (dyspareunia and dysuria). Medical workup has been negative for evident pathology. Her primary care physician has tried opiate analgesics, which were temporarily relieving, but the patient’s pain complaints persisted, and she became dependent on the medications. She endorses some sadness and frustration that “no one has been able to help me” and she also meets full criteria for depression.

**Venlafaxine (Effexor)**

Patient is a 70-year-old female with Type-2 diabetes and meets full criteria for major depression. She also complains of chronic back pain and diabetic peripheral neuropathy. She is in your psychiatric office and asking for assistance.

**Bupropion (Wellbutrin)**

Patient is a 36-year-old male with a history of depression and inattention, who found SSRIs unhelpful due to sexual dysfunction. He meets full criteria for major depression and is interested in medication to help him.

**Gabapentin (Neurontin)**

Patient is a 60-year-old female with mild, daily worrying/anxiety, who also has difficulty with post-herpetic facial neuralgia in the distribution of the right Trigeminal nerve. She is afraid of taking “sleeping pills” and opiate pain medication, and does not meet criteria for depression. Her PCM suggested she consider gabapentin and sent her to you for a psychiatric assessment and re-consideration of gabapentin for her pain and anxiety.

**Informed Consent Exercise Week 2**

**Disulfiram (Antabuse)**

Patient is a 34-year-old man who presents to your primary care office with vague complaints of a headache. He acknowledges that he has been struggling lately—that his job performance as a salesman has been poor of late and that he and his wife are talking about separating. He also cites legal concerns about a recent DUI charge. He is able to acknowledge that alcohol is at the root of many of his problems and asks for help to stop drinking.

**Mirtazapine (Remeron)**

Patient is a 65-year-old male with a history of significant life-long depression and anxiety, who tried SSRIs in the past with complaints of agitation, sleeplessness and sexual dysfunction due to the medications. He presents with complaints of anxious depression, initial and middle insomnia, as well as poor appetite with weight loss. Medical work-up is non-contributory.

**Zolpidem (Ambien)**

Patient is a 48-year-old woman with a history of rheumatoid arthritis who complains that she has been having a lot of difficulty with sleep for the last several months, especially since her most recent RA flair. Even though her pain has subsided generally, she continues to have problems falling asleep and wakes up several times during the night. She denies depressive symptoms currently or in the past and has no history of substance misuse.

**Lorazepam (Ativan)**

Patient is a 26-year-old woman with a past medical history of endometriosis who presents with symptoms of acute panic including intense fear, tachycardia, shortness of breath and nausea when doing presentations at work. She requests help, but does not want to be on frequent, daily medication—only as needed.

**Eszopiclone (Lunesta)**

Patient is a 52-year-old woman with h/o hypothyroidism, recently diagnosed with obstructive sleep apnea. She was referred to you to assist with treatment of her insomnia and irritability, worsened by her new CPAP machine that is also disrupting her sleep.

**Informed Consent Exercise Week 3**

**Haloperidol (Haldol)**

Patient is a 21-year-old man who was diagnosed with schizophrenia in his late teens. He was put on risperidone initially but gained some weight, so stopped that medication. Currently, he hears voices several times a day that say negative things about him, and he continues to be worried that the police have bugged his house. He works the night shift at a convenience store, but has no health insurance currently, and is very concerned about the cost of his medication.

**Olanzapine (Zyprexa)**

Patient is a 19-year-old enlisted soldier who was admitted to the base hospital with a recent onset of bizarre behavior including barricading himself in his barracks. He thinks that the military police are spying on him, his food is poisoned, and he seems to be fairly disorganized in his thought process. He has lost 15 pounds since his enlistment. Since admission, he has been bizarre and largely non-communicative with staff.

**Clozapine (Clozaril)**

Patient is a 25-year-old man with a history of schizophrenia that has been largely refractory to prior treatment with risperidone, ziprasidone, olanzapine and perphenazine. He has been readmitted to the hospital with auditory hallucinations commanding him to jump off a bridge. He is somewhat overweight and smokes cigarettes but is otherwise physically healthy. He has family and community support.

**Aripiprazole (Abilify)**

Patient is a 33-year-old woman with a history of depression and diabetes mellitus who has experiencing worsening mood symptoms for the last several months. She currently endorses significant dysphoria and anhedonia. She has recently developed the thought that her internal organs are shrinking and is concerned that she will not only not be able to have another child but also that she is going to “die on the inside.” Her dose of fluoxetine was increased last week from 20mg to 60mg daily, but she has yet to see any effect.

**Quetiapine (Seroquel)**

Patient is a 38-year-old male with a hx of military trauma (lost 3 friends in IED blast where he assisted in recovery of remains) associated with significant initial and middle and early morning insomnia with vivid nightmares, flashbacks, social withdrawal, poor appetite, and morbid fascination with his own death and possible suicide. He is on a therapeutic dose of sertraline for depression. He could not tolerate prazosin for nightmares.

**Informed Consent Exercise Week 4**

**Donepezil (Aricept)**

Patient is a 79-year-old woman with a 1-year history (per family) of memory loss. The patient largely denies any problems, simply saying, “I don’t get around as well as I used to,” but says she has not noticed any specific concerns about her memory. She has a history of vaginal cancer (resected) and takes only ASA. Her MMSE score is 22/30, down from 27/30 one year ago. Her family reports that she is unable to keep up with finances and occasionally gets lost on previously familiar routes.

**Memantine (Namenda)**

Patient is an 85-year-old man with a 5-year history of worsening memory and other symptoms including having trouble getting dressed appropriately and some word finding difficulty. His MMSE score is 18/20. He is pleasant and cooperative but seems to minimize any difficulties: “I’m fine except for my knees.” He has a history of hypertension, coronary artery disease and peripheral vascular disease and takes aspirin and enalapril.

**Risperidone (Risperdal)**

Patient is an 83-year-old man with a long-standing diagnosis of dementia who resides in a skilled nursing facility. He has a tendency to become agitated at times, particularly at night. A previously genial and gentle man, he has recently been yelling at fellow residents and has punched several members of the staff while they are assisting him. Trials of melatonin, trazodone and other antidepressants, 1:1 supervision has been unhelpful, and a full medical assessment for infectious/metabolic causes of agitation have been negative.

**Melatonin**

Patient is a 70-year-old female without medical problems except for a 4-year history of memory difficulties relatively well managed with donepezil and memantine is in your office complaining of difficulty falling asleep and maintaining sleep. Her live-in family reports some mild increased irritability just before bedtime.

**Trazodone**

Patient is a 78-year-old male complains of disturbed and unrestful sleep, with normal sleep study and no medical problems. He has trouble falling asleep and remaining asleep. He has no early morning awakening. He also complains of mild general anxiety without panic, decreased appetite, somewhat depressed mood associated with increased daytime irritability, but without reduced concentration, interest, and suicidal or morbid thoughts. He has a friend on trazodone, and wonders if it may help him.

**Informed Consent Exercise Week 5**

**Lithium**

Patient is a 28-year-old woman with a history of depression who presents with symptoms concerning for mania—racing thoughts, decreased need for sleep, uncharacteristic aggression toward her husband and a recent spontaneous purchase of a new car. She had taken citalopram until one year ago for prior symptoms of depression but is not currently on any medication.

**Valproic Acid (Depakote)**

Patient is a 24-year-old man with a history of prior manic episodes who was admitted to the psychiatric ward for increasingly reckless behavior including racing his car over 100 mph around the beltway and then resisting arrest, including claiming to be “the Messiah of Maryland.” Since admission he has continued to be extremely irritable and grandiose but is willing to consider medication if taking it will let him leave the hospital.

**Methylphenidate (Concerta)**

Patient is an 8-year-old boy who is brought into your pediatrics office by his parents at the request of his teachers. He is “bright” and “sweet”, but frequently gets into trouble at school because of an inability to sit still and frequent disruptions of class. His parents agree that he has a lot of energy and some difficulty staying on tasks but are also very invested in his athleticism and are concerned that he continues normal physical development.

**Lamotrigine (Lamictal)**

Patient is a 30-year-old man with a history of bipolar disorder including 2 hospitalizations for mania who presents complaining of feelings of worthlessness, helplessness and suicidal ideation, which have worsened over the last month. He took olanzapine and lithium during his last hospitalization but stopped after his mood stabilized after discharge because “I was getting fat.” He reports a few prior depressive episodes but none this severe. A previous trial of paroxetine “caused me to go crazy.”

**Electroconvulsive Therapy (ECT)**

Patient is a 45-year-old male lawyer with severe depression, which has been resistant to treatment with MAOIs, Lithium, SSRIs and SNRIs. His family is concerned that he is too depressed to go to work, eat, or concentrate. He presents with poor eye contact, few verbalizations, obviously malnourished, and on exam has “waxy flexibility” with normal vital signs. You decide to discuss ECT as a treatment option with the patient and his wife.
