# Supplementary material for: Psychotropic Medication Informed Consent: A Cross-Specialty Role-Playing Skill Builder
Source: MedEdPORTAL. 2021 May 5;17:11152. doi: 10.15766/mep_2374-8265.11152 (PMC8096884; doi:10.15766/mep_2374-8265.11152)
Supplement: Supplementary file 1 — Student Instructions.docxVignettes.docxIC & Medication Study Card Instructions.docxFaculty Instructions.docxPeer & Supervisor Feedback Form.docxExample.mp4Essential Elements of Communication.pdfStudent Survey.docx [file mep_2374-8265.11152-s001.zip › C. IC & Medication Study Card Instructions.docx]

**Informed Consent Exercise**

**Medication Study Card Instructions**

For each week, you should use the following rubric and develop Medication Fact Cards in this format. You will need to look up the following facts for five medications **before** you get to class. Items that you should include on review cards:

- Indication(s): *Why are you recommending this medication for this patient?*
- Mechanism of Action: *Learn mechanism at physician level, and prep words for patients to avoid jargon*
- Benefits: “*This will help reduce the XYZ symptoms you are experiencing.”*
- Contraindications: Things to ask about before prescribing: *Do you have liver failure?*
- Duration of Use: *Am I going to be on this forever, doc?*
- Dosing Schedule: *Twice a day? Once a day? As needed?*
- Common/Expected Side Effects and Workarounds: “*Everybody gets an upset tummy but call if XYZ.”*
- Black Box Warnings: *Best to hear these from a doctor first, not the Internet.*
- Possible Toxicities/Adverse Drug Reactions (ADRs): *Things worse than expected side effects (especially “special” stuff for this specific med)*
- Alternate treatments/other meds available: *Other meds, ECT, talk therapy?*
- Course of Illness with and without medication: *One month versus one year?*
- Follow-up: “*Let’s see you back in 3 days/1 week/2 weeks” (or whenever is appropriate for that med)*
- Emergency Contact:  *When/how a patient should contact you or clinic, and what problems to call for.*
