# Supplementary material for: Psychotropic Medication Informed Consent: A Cross-Specialty Role-Playing Skill Builder
Source: MedEdPORTAL. 2021 May 5;17:11152. doi: 10.15766/mep_2374-8265.11152 (PMC8096884; doi:10.15766/mep_2374-8265.11152)
Supplement: Supplementary file 1 — Student Instructions.docxVignettes.docxIC & Medication Study Card Instructions.docxFaculty Instructions.docxPeer & Supervisor Feedback Form.docxExample.mp4Essential Elements of Communication.pdfStudent Survey.docx [file mep_2374-8265.11152-s001.zip › D. Faculty Instructions.docx]

**Informed Consent Exercise**

**Faculty Instructions**

Objectives for Learners:

1. Memorize and utilize an essential steps rubric for obtaining Informed Consent

2. Develop and demonstrate psychotropic medication fund of knowledge

3. Propose and discuss medication plan in patient-friendly terms

4. Practice shared decision-making techniques

5. Assess and discuss self and peer performance in obtaining informed consent

This exercise focuses on specific aspects of obtaining informed consent: presenting information about the recommended treatments and engaging in shared decision making. Please reinforce with your learners that the entire informed consent process involves assessing patient understanding, sharing information about the recommended treatment(s), engaging in shared decision-making, and documenting the discussion.

The benefits of this exercise are that students have the opportunity to try on styles and approaches to get the words out (it’s not about “being right”). Students learn high-yield drug facts in preparation for this exercise and faculty get to review/relearn/update drug facts, too! For the first few weeks, consider writing the rubric on the board and having a student scribe in order to make a completed drug fact sheet together as a class.

**BEFORE ROLE-PLAY:**

- Allow 5 min prep (in case students did not do their homework!)
- Review the example video informed consent together
- Ask for volunteer “patient” and “physician” for role-play…or mix it up, and assign some weeks or volunteer others…and for real fun—have faculty play the patient
- Set time limit: start with 15 minutes, then get faster as rotation progresses
- Keep level of learner in mind (first clerkship rotation or PGY1?)

**DURING ROLE-PLAY:**

- During the first exercise, have faculty demonstrate obtaining Informed Consent as the physician…model what you do!
- While the students are doing the role-play, listen, jot down a few notes/observations on Peer/Supervisor Feedback Form
- Allow students to break “fourth wall”, but encourage help to come from peers before faculty “rescues”
- Laugh and “get into” the role-play, however, referee/rein-in if students get too silly or too stern/rude

**AFTER ROLE-PLAY:**

- Ask students what they felt/did well/missed once the “physician” is done
- Allow peers to comment on what went well/needs improvement
- Chime in last with observations, ideas for new approaches, and medication fact corrections
- Create an Informed Consent “script” with students on the board and allow students/residents to snap a photo of the finished “model”
- Encourage students to ask residents and other faculty to observe them in the “real” world
- Show your own pharmacology tools and how you (the faculty) look up drug facts
