# Supplementary material for: Psychotropic Medication Informed Consent: A Cross-Specialty Role-Playing Skill Builder
Source: MedEdPORTAL. 2021 May 5;17:11152. doi: 10.15766/mep_2374-8265.11152 (PMC8096884; doi:10.15766/mep_2374-8265.11152)
Supplement: Supplementary file 1 — Student Instructions.docxVignettes.docxIC & Medication Study Card Instructions.docxFaculty Instructions.docxPeer & Supervisor Feedback Form.docxExample.mp4Essential Elements of Communication.pdfStudent Survey.docx [file mep_2374-8265.11152-s001.zip › E. Peer & Supervisor Feedback form.docx]

**INFORMED CONSENT PEER/SUPERVISOR FEEDBACK**

| Directions: Document each observed behavior to help your classmates recognize their strengths and weaknesses when obtaining informed consent. Consider Essential Elements of Communication items as well as content during this process. |
| --- |
| The student discussed the following **INDICATION** for selected medication:  ____________________________________  ____________________________________  ____________________________________ |
| The student discussed **MECHANISM OF ACTION** of the selected medication:  ____________________________________  ____________________________________  ____________________________________ |
| The student discussed the following **BENEFITS**:  ____________________________________  ____________________________________  ____________________________________  ____________________________________  ____________________________________  ____________________________________ |
| The student discussed **RISKS/CONTRAINDICATIONS/BLACK BOX WARNINGS:**  ____________________________________  ____________________________________  ____________________________________ |
| The student discussed **DURATION OF USE/DOSING SCHEDULE**:  ____________________________________  ____________________________________  ____________________________________ |
| The student discussed **COMMON/EXPECTED SIDE EFFECTS:**  ____________________________________  ____________________________________  ____________________________________  ____________________________________  ____________________________________  ____________________________________ |
| The student discussed **ADVERSE RXNS/TOXICITIES:**  ____________________________________  ____________________________________  ____________________________________ |
| The student discussed **MONITORING** FOR SIDE EFFECTS AND TOXICITIES:  ____________________________________  ____________________________________  ____________________________________ |
| The student discussed **POSSIBLE ALTERNATE/ADDITIONAL TREATMENTS**:  ____________________________________  ____________________________________  ____________________________________ |
| The student discussed **COURSE OF ILLNESS WITHOUT TREATMENT**:  ____________________________________  ____________________________________  ____________________________________ |
| The student discussed **TIMING OF FOLLOW-UP:**  ____________________________________  ____________________________________  ____________________________________ |
| The student discussed **COURSE OF ACTION FOR QUESTIONS OR EMERGENCY:**  ____________________________________  ____________________________________  ____________________________________ |
