# Supplementary material for: Psychotropic Medication Informed Consent: A Cross-Specialty Role-Playing Skill Builder
Source: MedEdPORTAL. 2021 May 5;17:11152. doi: 10.15766/mep_2374-8265.11152 (PMC8096884; doi:10.15766/mep_2374-8265.11152)
Supplement: Supplementary file 1 — Student Instructions.docxVignettes.docxIC & Medication Study Card Instructions.docxFaculty Instructions.docxPeer & Supervisor Feedback Form.docxExample.mp4Essential Elements of Communication.pdfStudent Survey.docx [file mep_2374-8265.11152-s001.zip › G. Essential Elements of Communication.pdf]

USUHS Psychiatry Clerkship
Essential Elements of Communication

© 7/18/2005 University of New Mexico School of Medicine
Adapted with permission from paper format to fillable form and used with permission

Please use this checklist as a tool for prompting your rating of the student in each of the 8 above sections.

STUDENT NAME:

1. OPEN THE DISCUSSION ○ 1 ○ 2 ○ 3 ○ 4 ○ 5

	1	2	3	4	5
Introduction	○ No greeting<br>○ Does not call you by name<br>○ Initiates use of inappropriate variation of your name<br>○ Inappropriate familiarity or informality<br>○ Does not identify self by name or title		○ Polite greeting<br>○ Calls you by or establishes your appropriate name<br>○ Appropriate formality<br>○ Accurately introduces self with full name and title		○ Personal greeting shows genuine interest<br>○ Displays welcoming nonverbal behavior
Patient opening	○ Begins with closed-ended question<br>○ Interrupts your initial response		○ Begins with open-ended question<br>○ Does not interrupt your initial response		○ Asks if there is anything else you want to add after you finish your initial statement<br>○ Summarizes your opening concerns and verifies with you
Agenda setting	○ Offers no organizational overview regarding what to expect during the encounter		○ Offers an early, brief outline of what to expect<br>○ Does not verify the agenda with you		○ Offers timely, detailed outline of what to expect during the encounter<br>○ Verifies the agenda with you<br>○ Includes an agenda for subsequent visits

2. BUILD A RELATIONSHIP ○ 1 ○ 2 ○ 3 ○ 4 ○ 5

	1	2	3	4	5
Listening	○ Misunderstands what you say<br>○ Does not acknowledge or allow attempts to add or correct information<br>○ Frequently repeats questions<br>○ Interrupts your responses		○ Seems to understand what you say<br>○ Accepts correction<br>○ Uses previous information as basis for subsequent questions<br>○ Rarely interrupts<br>○ Summarizes at least once		○ Acquires and accurately assimilates the facts and subtleties of your situation<br>○ Does not interrupt important silences<br>○ Uses restatements, summaries, or explicit checks to verify information
Empathy & attitude	○ Gives false reassurance<br>○ Does not acknowledge your situation		○ Demonstrates or expresses appropriate concern for you		○ Responds appropriately to each of your concerns or issues<br>○ Provides nonjudgmental support<br>○ Helps you clarify your own feelings and thoughts<br>○ Expresses genuine concern throughout the encounter
Nonverbal behavior	○ Inappropriate or distracting behaviors<br>○ Inappropriately groomed, disheveled, malodorous<br>○ Unprofessional clothing or adornment<br>○ Distracted manner		○ Tone of voice, facial expression, posture, nodding, touch, and distance are appropriate<br>○ Makes appropriate eye contact<br>○ Professional and appropriate clothing or adornment		○ Tone of voice and facial expressions consistently indicate interest and concern<br>○ Uses receptive postures<br>○ Makes mutually agreeable adjustments in distance or touch for your comfort

3. GATHER INFORMATION ○ 1 ○ 2 ○ 3 ○ 4 ○ 5

	1	2	3	4	5
Context	○ Does not obtain any information about you as a person		○ Acquires sufficient information about you as a person<br>○ Seems interested in and briefly explores your life context		○ Acquires important information about you as a person<br>○ Encourages you to share freely your reasons for seeking medical attention
Questions	○ Rarely balances open- and closed-ended questions<br>○ Most questions are closed-ended<br>○ Questions seem mechanistic and rote		○ Balances open- and closed-ended questions appropriately<br>○ Uses closed-ended questions to check details		○ Questions are tailored to you as an individual<br>○ Prompts you to talk freely in response to open-ended questions<br>○ Clarifies specific information or details through closed-ended questions
Organization & transitions	○ Transitions are confusing and disorganized<br>○ Disconcerting, jarring, or random topic changes		○ Explains transitions<br>○ Occasionally backtracks to omitted or forgotten question		○ Transitions are seamless and smooth<br>○ Clear, logical transitions that may be explicit or implicit

4. UNDERSTAND THE PATIENT'S PERSPECTIVE ○ 1 ○ 2 ○ 3 ○ 4 ○ 5

	1	2	3	4	5
Patient concerns	○ Doesn't ask about your concerns<br>○ Ignores concerns you raise		○ Asks you to express your major concerns at some point in the interview<br>○ Follows up on concerns you raise explicitly		○ Elicits your major concerns early in the encounter<br>○ Consistently follows up on clues or information you volunteer
Patient beliefs & preferences	○ Does not elicit requests or expectations for outcomes today<br>○ Interrupts with suggestions before hearing your preferences<br>○ Denies or ignores your requests without explanation<br>○ Belittles your perspective		○ Elicits your beliefs or preferences<br>○ Addresses most of your requests<br>○ Acknowledges your perspective		○ Acknowledges your elicited beliefs and preferences<br>○ Consistently addresses your beliefs, preferences, and requests<br>○ Responds to your perspective as understandable and valid
Expression of feelings	○ Denigrates you<br>○ Becomes silent and withdrawn<br>○ Changes the subject when you express emotion		○ Recognizes and acknowledges explicit expression of emotions<br>○ Asks about your emotions after you have given clues		○ Facilitates the expression of your feelings<br>○ Anticipates emotional reactions you might be expected to have<br>○ Elicits your means of emotional support
(Consider for particularly vulnerable patient populations, e.g., patients who use another language, have dementia or mental illness, or have marked physical limitations that may require special accommodations.)					
Specific circumstances	○ Does not demonstrate awareness of unusual circumstances		○ Demonstrates awareness of unusual circumstances and makes accommodation		○ Makes attentive, respectful, resourceful, and effective accommodation for unusual circumstances

5. SHARE INFORMATION ○ 1 ○ 2 ○ 3 ○ 4 ○ 5

	1	2	3	4	5
Vocabulary	○ Uses language you don't understand<br>○ Uses inappropriate language (slang or technical)		○ Uses language appropriate to your educational or intellectual level<br>○ Clarifies vocabulary upon request		○ Checks your understanding of technical words and explains if necessary<br>○ Skillful use of technical vocabulary
Patient understanding of illness	○ Doesn't elicit your understanding of your illness or situation		○ Acknowledges when you volunteer your understanding of your illness or situation		○ Asks about your understanding of your illness or situation<br>○ Highlights areas of similarity between your understanding and medical science
Clinician information & explanation	○ Ignores your requests for information<br>○ Consistently disregards opportunities for instruction		○ Gives information that is specific and clear, but not personalized		○ Gives full, clear, and thorough explanation of what your symptoms might mean or how they could be investigated<br>○ Verifies your understanding of information<br>○ Offers to provide additional information

6. REACH AGREEMENT (Planning Evaluation and Treatment) ○ 1 ○ 2 ○ 3 ○ 4 ○ 5

	1	2	3	4	5
Negotiation	○ No plan<br>○ Presents a nonnegotiable plan		○ Presents a plan and requests feedback		○ Solicits input, negotiates a plan to the extent you desire, and confirms your understanding of the final plan
Implementation	○ Does not address your ability to implement the plan<br>○ Ignores or denigrates your ability to implement the plan		○ Addresses your hesitations, suggestions, or questions about implementing the plan<br>○ Assumes you are capable of implementing the plan		○ Elicits your suggestions or questions about implementing the plan<br>○ Explores barriers to implementing the plan and facilitates possible solutions

7. PROVIDE CLOSURE ○ 1 ○ 2 ○ 3 ○ 4 ○ 5

	1	2	3	4	5
Patient next steps	○ Stops abruptly<br>○ No indication of next steps (e.g. get dressed, wait in room, make another appointment, etc.)		○ Clear explanation of next steps		○ Verifies next steps with you (e.g. get dressed, wait in room, make another appointment, etc.)
Physician conclusion	○ No conclusion		○ Polite, generic conclusion		○ Polite, personalized, thoughtful conclusion

8. OVERALL RATING ○ 1 ○ 2 ○ 3 ○ 4 ○ 5

	1	2	3	4	5
	○ I would not return to this clinician under any circumstances		○ I would return to this clinician		○ I would return to this clinician above all others<br>○ I would want this clinician to care for all my loved ones

Comments:
