# Supplementary material for: Psychotropic Medication Informed Consent: A Cross-Specialty Role-Playing Skill Builder
Source: MedEdPORTAL. 2021 May 5;17:11152. doi: 10.15766/mep_2374-8265.11152 (PMC8096884; doi:10.15766/mep_2374-8265.11152)
Supplement: Supplementary file 1 — Student Instructions.docxVignettes.docxIC & Medication Study Card Instructions.docxFaculty Instructions.docxPeer & Supervisor Feedback Form.docxExample.mp4Essential Elements of Communication.pdfStudent Survey.docx [file mep_2374-8265.11152-s001.zip › H. Student Survey.docx]

Informed Consent Exercise Survey

Dear Students,

The Psychiatry Clerkship asks for your feedback on Informed Consent exercises you completed during the weekly didactics. Completing this feedback form takes approximately 2 minutes and is voluntary. Information gathered will help inform future development of this section for subsequent rotations. Please us know if you have any questions.

1. Where did you complete your psychiatry clerkship rotation?

*Mark only one checkbox.*

NCA site.

Non-NCA site.

1. Please let us know how much you agree/disagree with each statement

*Mark only one box per row.*

|  | **Strongly Disagree** | **Disagree** | **Neutral** | **Agree** | **Strongly Disagree** | **N/A** |
| --- | --- | --- | --- | --- | --- | --- |
| I was familiar with the most of elements of informed consent prior the clerkship exercises. |  |  |  |  |  |  |
| Other rotations had similar exercises to teach informed consent. |  |  |  |  |  |  |
| The medications/procedures selected for the exercises were appropriate. |  |  |  |  |  |  |
| I prepared for the informed content exercise prior to class. |  |  |  |  |  |  |
| I performed an informed consent while on my psychiatry clinical service. |  |  |  |  |  |  |
| This exercise increased my comfort in obtaining informed consent while on the psychiatry service. |  |  |  |  |  |  |
| Exercise increased my comfort in obtaining medication informed consent while on another clinical service. |  |  |  |  |  |  |
| Exercise and preparation for the exercise helped with studying for the Psychiatry NBME. |  |  |  |  |  |  |
| Exercise and preparation for the exercise helped with studying for the Psychiatry OSCE. |  |  |  |  |  |  |

1. What were the strengths of the medication/procedure informed consent exercise?

1. What would you suggest as improvements for the informed consent exercise?
